# Supplementary material for: Netcombin: An algorithm for constructing optimal phylogenetic network from rooted triplets
Source: PLoS One. 2020 Sep 18;15(9):e0227842. doi: 10.1371/journal.pone.0227842 (PMC7500971; doi:10.1371/journal.pone.0227842)
Supplement: S4 File — (ZIP) [file pone.0227842.s004.zip › SequenceData-Triplet/sequenceData-10-20-30-0-Triplet/length 1000, numseq 40, rate 10^-7/3 seq.pdf]

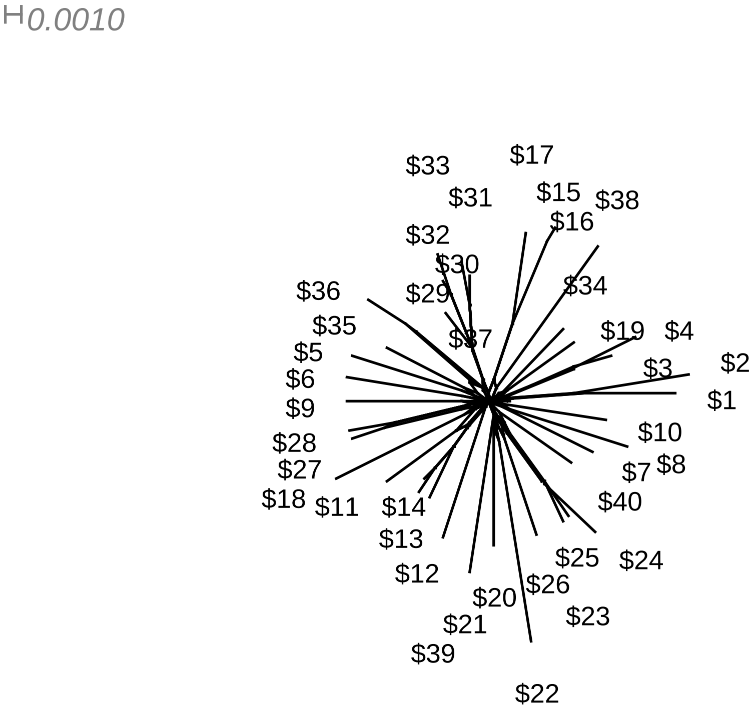

0.0010
$17
$33
$31
$15
$38
$16
$32
$30
$34
$36
$29
$19
$4
$35
$37
$5
$2
$3
$6
$1
$9
$10
$28
$8
$7
$27
$18
$40
$11
$14
$13
$25
$24
$12
$26
$20
$23
$21
$39
$22
